# A retrospective, descriptive study of hepatitis C testing, prevalence, and care continuum among adults on probation

**DOI:** 10.1186/s40352-022-00191-9

**Published:** 2022-08-10

**Authors:** Kevin F. Kamis, David L. Wyles, Matthew S. Minturn, Tracy Scott, Dean McEwen, Hermione Hurley, Scott J. Prendergast, Jessie Gunter, Sarah E. Rowan

**Affiliations:** 1 Public Health Institute at Denver Health, Denver Health and Hospital Authority, 601 Broadway, 8th floor, MC 2800, Denver, CO 80203-3407 USA; 2 Division of Infectious Diseases, Denver Health Medical Center and University of Colorado School of Medicine, Aurora, CO USA; 3 Department of Medicine, University of Colorado School of Medicine, Aurora, CO USA; 4 LGBTQ+ Health Services, Denver Health and Hospital Authority, Denver, CO USA; 5 Center for Addiction Medicine, Denver Health and Hospital Authority, Denver, CO USA; 6 Denver Adult Probation, Colorado Judicial Branch, Denver, CO USA; 7 Colorado Department of Public Health & Environment, Denver, CO USA

**Keywords:** Hepatitis C virus, Criminal justice, Adult probation, Public health

## Abstract

**Background:**

Despite constituting the largest segment of the correctional population, individuals on court-ordered probation remain largely unstudied with respect to hepatitis C virus (HCV) testing and linkage-to-care. We conducted a retrospective, descriptive analysis to estimate prevalence of diagnosed HCV and the subsequent HCV care cascade among a cohort of individuals enrolled in an adult probation program over a 25-month period in Denver, Colorado.

**Methods:**

We utilized probabilistic matching with first and last name, sex, and birthdate to identify individuals enrolled in probation between July 1, 2016 and July 30, 2018 who had a medical record at the participating safety-net healthcare institution as of December 31, 2019. Electronic medical record data were queried for evidence of HCV testing and care through June 30, 2021. The state HCV registry was also queried for prevalence of reported HCV cases among the cohort.

**Results:**

This cohort included 8,903 individuals; 6,920 (78%) individuals had a medical record at the participating institution, and of these, 1,037 (15%) had ever been tested for HCV (Ab or RNA) and 308 (4% of those with a medical record, 30% of those tested) had detectable HCV RNA. Of these, 105 (34%) initiated HCV treatment, 89 (29%) had a subsequent undetectable HCV viral load, and 65 (21%) had documentation of HCV cure. Eleven percent of the total cohort had records of positive HCV Ab or RNA tests in the state HCV registry.

**Conclusions:**

This study demonstrates the importance of HCV screening and linkage-to-care for individuals enrolled in probation programs. A focus on this population could enhance progress towards HCV elimination goals.

## Background

Within the criminal justice system, probation is a sentence in which an individual convicted of a crime remains in the community under court-ordered supervision rather than serving a prison sentence. Those on probation comprise the largest single group of the US correctional population, exceeding 3.5 million individuals in 2018 and outnumbering those in jails and prisons by more than 1.3 million (Bureau of Justice Statistics, 2021). Individuals on probation have a higher burden of physical health conditions, mental illness, and substance use disorders compared to the general population (Hawks et al., 2020). One study found that persons on probation had higher age-standardized mortality compared to those in jail, prison, or the general population (Wildeman et al., 2019). Nonetheless, there remains a dearth of health-related research centering individuals on probation compared to individuals incarcerated in prisons or jails (Hawks et al., 2020; O'Connell et al., 2020; Wildeman et al., 2019; Zaller et al., 2016). One area of high public health importance meriting additional research is hepatitis C among the probation population.

Incidence of acute HCV has increased in recent years driven primarily by injection drug use (Centers for Disease Control and Prevention Division of Viral Hepatitis National Center for HIV/AIDS Viral Hepatitis STD and TB Prevention, 2021). HCV-associated mortality surpassed that of all other national notifiable infectious diseases in 2012 (Ly et al., 2016) and remains high (Ly et al., 2020). Black, Indigenous, and Latinx/Hispanic populations are disproportionately impacted by the hepatitis C epidemic in the United States (Bradley et al., 2020; Centers for Disease Control & Prevention, 2021b; Denniston et al., 2014). An estimated 2.4 million individuals in the United States currently have HCV (Hofmeister et al., 2019) and in the era of curative and well-tolerated direct-acting antivirals (Falade-Nwulia et al., 2017; Younossi & Henry, 2015), the medical and public health communities now have the biomedical tools to eliminate HCV. However, of all those in the United States to have ever had HCV, only 52% are estimated to be aware of their infection and 37% to be cured (Chhatwal et al., 2019).

Individuals incarcerated in prisons or jails are known to bear a disproportionate burden of HCV, representing an estimated 29% to 33% of all HCV in the US (Varan et al., 2014). Only 4% of the estimated 1.34 million individuals with HCV who are incarcerated are thought to have been cured (Chhatwal et al., 2019). Focused efforts on the correctional system are therefore needed to target testing and linkage to care among populations at increased HCV risk (Rich et al., 2014). Despite comprising the largest portion of the criminal justice system, the probation population has been largely understudied in regard to HCV prevalence, engagement in care, and treatment (Martin et al., 2003; Zaller et al., 2016). Some studies have assessed HCV prevalence among this population, including a study that found 9% (12/130) HCV antibody positivity among a group of individuals on probation or parole in Rhode Island (Zaller et al., 2016) and a national survey that found a nearly five-fold higher adjusted odds ratio of having hepatitis C or hepatitis B among probationers compared to the general population (Hawks et al., 2020). Our group conducted a study of HCV testing located on-site at a probation department where we found a 13% HCV antibody positivity among the 417 individuals tested (Kamis et al., 2022).

To expand the evidence of hepatitis C prevalence among individuals on probation, we conducted a retrospective, we conducted a retrospective, descriptive analysis to estimate prevalence of diagnosed HCV among a cohort of individuals in probation programs over a 25-month period in Denver, Colorado. We established a care cascade for the subset of anti-HCV positive individuals on probation with established medical care in the local safety-net healthcare system.

## Methods

### Settings

Denver Adult Probation supports the District Court of the 2nd Judicial District for the State of Colorado and provides supervision and related services to all adult-aged individuals sentenced to felony probation who reside within the judicial district. At any given time, Denver Adult Probation serves between 4,500 and 5,000 clients in several programs tailored to meet their specific needs.

Denver Health and Hospital Authority (hereafter Denver Health) is an integrated, public safety net institution including a 555-bed acute care hospital, nine federally qualified community health centers, and several specialty clinics, two of which provide HCV evaluation and treatment. Denver Health patients are screened for HCV per the most recent US Preventive Services Task Force recommendations (U. S. Preventive Services Task Force, 2020), provider discretion, or patient request.

### Study population

Individuals 18 years of age and older who entered Denver Adult Probation between July 1, 2016 and July 30, 2018 were included in this analysis.

### Data analysis

We conducted a retrospective, descriptive study in order to describe the HCV care continuum of adults on probation who sought care at the local safety net health system. We used probabilistic matching using first name, last name, sex, and birthdate to identify individuals from the probation cohort who had a medical record at Denver Health. The Denver Health electronic medical record was then queried for evidence of HCV testing at Denver Health between April 9, 2016 (the date the current electronic medical record system launched at Denver Health) and December 31, 2019. Records for individuals with positive HCV antibody (Ab) or RNA were reviewed via chart review for progression through the HCV treatment cascade through June 30, 2021.

To estimate the total number of individuals among the probation cohort tested for HCV including those outside of the Denver Health system but within the state of Colorado, the Colorado Electronic Disease Reporting System (CEDRS) was queried on December 3, 2019 by the Colorado Department of Public Health & Environment for aggregate number of HCV cases. The state registry dates back to 1993 and includes cases of chronic or acute HCV with a status of probable or confirmed HCV case as defined by the Council of State and Territorial Epidemiologists (CSTE) of the CDC (Centers for Disease Control & Prevention, 2021a).

Selection of cut off dates allowed for assessment of HCV testing both prior to and after entering probation as well as for progression through the care cascade past the period of probation. Exact dates of these pulls were subject to the practical limitations of coordinating data pulls and matching between three distinct systems: the local safety net health system, the state health department, and a municipal probation department.

Individuals tested for HCV on-site at the probation office through community-based outreach testing via a Denver Health/Denver Adult Probation partnership that launched September 5, 2018 were excluded. The results of this study are published elsewhere (Kamis et al., 2022).

### Ethics approval and consent to participate

All aspects of this study were approved by the Colorado Multiple Institutional Review Board. Informed consent was not obtained as this was a retrospective study. Judicial data were requested through a Data Request pursuant to CJD 05–01 Sect. 4.40(f): Colorado Judicial Department Public Access Policy.

## Results

A total of 8,903 individuals 18 years of age and older were enrolled in Denver Adult Probation Programs between July 1, 2016 and July 31, 2018; 76% were male. Median age as of July 31, 2018 was 33 years (Interquartile range [IQR]: 27—41). Nearly 10% of the entire probation cohort (858/8,903) were born between the years 1945 and 1965, falling into the *baby boomer* cohort, a group at increased risk for HCV as defined by the Centers for Disease Control and Prevention (Smith et al., 2012).

**Fig. 1:**
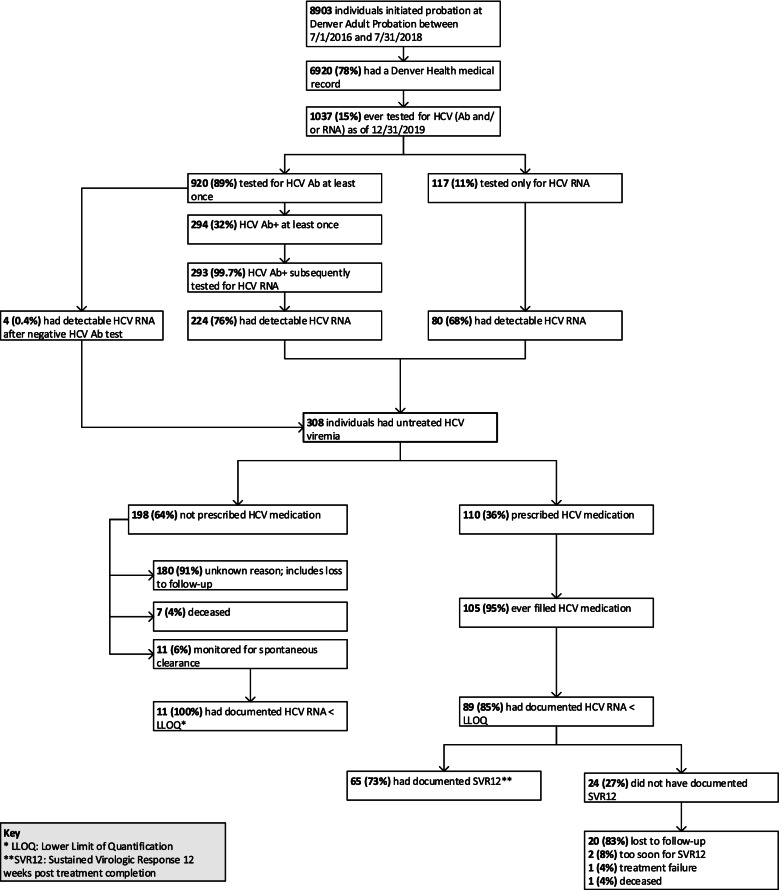
Hepatitis C virus testing and care continuum of a historical cohort of adults on probation

Of those on probation during this time period, 6,920 (78%) had a Denver Health Medical record as of December 31, 2019 and 1,037 (15%) had evidence of past HCV Ab or HCV RNA testing (Fig. 1). Of the 920 individuals who were tested for HCV Ab at least once, 294 (32%) were anti-HCV Ab positive and 224 (76%) of the 293 with subsequent RNA testing had detectable HCV RNA. Four additional individuals had detectable HCV RNA after previous negative HCV Ab tests. A total of 308 individuals had HCV RNA detected through testing at Denver Health in this cohort. The majority (74%) were male, median age at time of the HCV RNA test was 37 years (IQR: 30—50 years), 68% were non-Hispanic white, 12% non-Hispanic Black, and 19% Hispanic/Latinx of all races (Table 1). Nearly 80% were insured by Medicaid.

**Table 1:** Characteristics of adults supervised by probation program testing positve for HCV RNA at Denver Health

Metric	N (%) or Median(IQR)
Total	308 (100)
**Sex**^**a**^	
Male	228 (74)
Female	80 (26)
**Age (years) at time of first detectable HCV RNA**	37 (30–50)
**Race/Ethnicity**^**b**^	
White, non-Hispanic/Latinx	207 (68)
Black, non-Hispanic/Latinx	36 (12)
Hispanic/Latinx	58 (19)
Other	4 (1)
**Health Insurance Coverage**^**c**^	
Medicaid	244 (79)
Medicare	22 (7)
Commercial	13 (4)
Uninsured	7 (2)
Correctional Care	21 (7)
Other	1 (0.3)

^a^Sex at birth, may not reflect current gender identiy

^b^Three individuals missing race/ethnicity data in electronic medical record

^c^Captured upon Denver Health medical record query, may not be reflective of health insurance coverage status(es) when an individual was being followed by Denver Adult Probation

As of June 30, 2021, 110 (36%) of the 308 individuals with HCV viremia had been prescribed HCV medication, and 105 (34%) had filled at least one prescription. Of those who filled at least one prescription, 89 (85%) had a subsequent HCV RNA level below the lower limit of quantification (LLOQ), and 65 (62%) had documented cure as indicated by sustained virologic response 12 weeks after treatment completion (SVR12) as of June 30,2021 (Fig. 1). One HCV recurrence was documented among those treated.

According to the Colorado Department of Public Health & Environment HCV registry, 947 individuals (11%) of the study cohort had reports of HCV events as defined by a positive Ab and/or detected HCV RNA in as of December 3, 2019. Of the 947 HCV events, 300 (32%) of the HCV reports occurred during the 25-month period of probation starts (July 2016 through July 2018) and 589 (62%) occurred before this period. The remaining 58 cases (6%) were identified in the registry after this period.

## Discussion

Data indicate that a minority of countries are on trajectories likely to accomplish the World Health Organization goals set forth for eliminating viral hepatitis as a major public health threat by 2030 (World Health Organization, 2016). Particularly in the US, the incidence of new infections has steadily increased over the last decade and attainment of 2030 HCV elimination goals is not expected before 2050 (Razavi et al., 2019). The COVID-19 pandemic has disrupted HCV care and is predicted to further impede progress of HCV elimination goals (Barocas et al., 2021). A shift in focus to so-called “micro-elimination” has arisen for specific populations with unique transmission or disease characteristics for whom meeting 2030 goals for HCV elimination may be attainable (Lazarus et al., 2017).

Persons incarcerated within the correctional system have been prioritized for micro-elimination efforts due to high HCV prevalence and interaction with a relatively coordinated (at least at the state level) system which can offer testing and treatment or services for HCV (Rich et al., 2014). Accordingly, national hepatitis C guidelines recommend opt-out HCV testing in jails and prisons and recommend treatment of HCV infection in prisons and jails if the sentence is sufficiently long to complete the recommended treatment course (American Association for the Study of Liver Diseases & Infectious Diseases Society of America, 2021; Federal Bureau of Prisons, 2018). However, current guidelines do not include testing or treatment recommendations for individuals on probation, the largest segment of the US correctional population, and HCV-focused research in this group remains limited.

Our data retrospectively assess HCV screening and linkage to care among a large probation population. We found only 15% of the population with Denver Health medical records had evidence of screening for HCV; yet of those screened, 30% had positive HCV RNA tests indicating current infection.

Data reported to the Colorado Department of Public Health & Environment indicate that nearly 11% (947) of the total probation cohort had reported confirmed or probable HCV infection, and of these cases, 62% (589) were reported prior to the period of probation starts. A significant number of individuals therefore start probation with untreated HCV or contract HCV while on probation. However, the number tested and thus the true prevalence of infection is unknown as negative tests are not reportable. True prevalence of HCV among the probation cohort is likely to be more than 11%.

Our results align with other studies which have shown a higher prevalence among adults on probation compared to the general population (Hawks et al., 2020; Zaller et al., 2016). We recently published results of a prospective HCV testing and linkage to care program located on-site at the same probation department of this retrospective analysis (Kamis et al., 2022). In that study, 13% (56/417) of individuals tested anti-HCV positive, and of the 34 individuals who were found to have detectable HCV RNA, 14 (41%) linked to care and 1 (3%) achieved cure. This study is notably different from this retrospective analysis in that it prospectively assessed on-site testing and linkage to care in the setting of an incentivized ($25) research study. However, both studies reinforce the finding of a higher HCV prevalence among the probation population compared to the general population and the need for enhanced efforts around linkage to and retainment in HCV care through cure as well as the potential benefit of alternative treatment models such as on-site treatment or telehealth.

Our study assessed testing from 2016–2019, during which time HCV screening was recommended for all those born from 1945–65 (Smith et al., 2012) as well as for any person with other risks for HCV infection such as drug use, HIV, transfusion before 1992 or multiple sexual partners (CDC, 1998). While we did not have data to determine HCV risk for individuals on probation, many were on probation for drug related offenses, and it is suspected a large proportion of individuals on probation would have had an indication for HCV screening at the time. CDC and USPSTF recommendation have since been updated to call for a one-time HCV screening test for all adults regardless of risk (Schillie et al., 2020).

Encouragingly, the majority (78%) of individuals in Denver Adult Probation had a Denver Health medical record indicating that our safety net health system is well positioned to test and treat many individuals with HCV in the Denver Adult Probation program. Only 2% of individuals were uninsured with nearly 80% insured by Medicaid. Though Medicaid coverage for HCV treatment varies by state, many states have eased restrictions including the removal of fibrosis and sobriety requirements (National Viral Hepatits Roundtable & Center for Health Law and Policy Innovation of Harvard Law School, 2021). This suggests that at least in states with expanded Medicaid coverage, insurance coverage for HCV medications is not likely to be a major barrier for being linked to and receiving care for HCV.

In our study, a large drop off exists between having a detectable HCV RNA test and being prescribed medications. Importantly, this includes individuals that were never seen by an HCV treatment provider. However, the majority of those prescribed HCV medications in our study progressed to undetectable HCV RNA viral loads and had evidence of cure. Other studies of HCV testing and linkage to care in jail (Akiyama et al., 2019; Beckwith et al., 2016; Shoenbachler et al., 2016) and probation and parole settings (Zaller et al., 2016) have also found that only a small proportion of those with HCV progress from testing through cure. Innovative care delivery methods are needed to help bridge this gap, particularly methods that engage individuals with lived experience within the US justice system (Wurcel et al., 2021). One promising next step would be to explore offering HCV treatment on-site at probation departments, a model that has been explored in other community venues such as syringe service programs (Eckhardt, Scherer, Winkelstein, Marks, & Edlin, 2018; Morris et al., 2017; Winetsky et al., 2020). Shifting HCV treatment to community-based non-specialist providers has been shown to be safe and effective (Kattakuzhy et al., 2017; Radley et al., 2019) and should also be considered, potentially coupled with on-site health navigation which one study found was significantly associated with linkage to primary care from a probation office (O'Connell et al., 2020). Lastly, simplified treatment models that reduce the number of required medical visits and pre-treatment laboratory testing have been successful and could advance HCV elimination at the population level (Solomon et al., 2022).

This study has limitations. First, we are unable to establish a temporal relationship between dates of HCV testing and treatment with dates of probation entrance and termination. These data would inform the impact of probation supervision on access to HCV screening and treatment. The cohort included individuals who started probation within a two-year period and was evaluated for testing and treatment over the same time periods. As such, individuals who entered probation earlier had a longer period with which to be tested and treated for HCV. Due to challenges of aligning data pulls across distinct systems, data pulls may have omitted individuals enrolled in the probation who had subsequent positive hepatitis C testing. Due to additional data limitations, we are unable to ascertain the HCV infection risk profile of individuals with Denver Health medical records and therefore are unable to estimate the number of missed opportunities for HCV testing. Furthermore, we are unable to construct an HCV care continuum for individuals seeking care at institutions outside of Denver Health. Our cascade therefore likely underrepresents the true number of individuals in the cohort who received HCV testing and follow-up care. This analysis did not measure the number of individuals who attended an appointment with an HCV treatment provider but rather utilized pharmacy fill data. This analysis did not examine impact of incarceration on HCV testing and treatment among this population on probation, however there was relatively little to no hepatitis C testing and treatment at jail and prison facilities in Colorado during the time periods of this analysis. We were unable to capture if documented sex at birth varied from current gender identity, preventing the ability to describe HCV positivity among transgender and other gender-expansive individuals. Lastly, Denver Adult Probation and Denver Health are less than two miles apart. Probation departments located further away from the closest HCV care provider may face additional challenges in successfully linking individuals to care.

This study demonstrates the importance of directing increased efforts around HCV testing and linkage to care among the adult probation population. There exists a well-established understanding that individuals involved in the justice system bear a disproportionate burden of HCV, yet the largest component of the US criminal justice system remains largely underserved regarding HCV testing and linkage to care. Individuals on probation live in the community and are required to report for their probation supervision requirements, offering the potential to engage individuals for HCV testing and support regularly. The US probation system also disproportionately impacts young men of color (Phelps, 2017), and addressing HCV among the probation population could therefore have crucial implications for addressing racial and ethnic health inequities of the US HCV epidemic.

## Conclusion

In this study, 6,920 of 8,903 adults on probation over a 25-month period had a medical record at the local safety net health system. Of those with a medical record, 1,037 were ever tested for hepatitis C (HCV) within the health system, 308 individuals were found to have detectable HCV RNA, 105 filled HCV medication, 89 had subsequent negative HCV RNA viral loads, and 65 individuals had documented sustained virologic response indicative of cure. A focus on the probation population could bolster progress towards HCV elimination goals and improve the health of many individuals on court-ordered probation. This requires collaboration between health systems, public health entities, and probation systems, including collaboration around data sharing which could potentially benefit clients by reducing redundancy and time and improve systems-level coordination. Such partnerships could create clear and expedient pathways through which adults initiating and currently on probation are linked to HCV care. These efforts could be extremely impactful when addressing this large public health priority.

## Data Availability

The datasets generated and analyzed during the current study are not publicly available because they contain protected health information and criminal justice-related data, but deidentified data are available from the corresponding author on reasonable request.
